# Supplementary material for: Avoiding Catastrophe: Active Dendrites Enable Multi-Task Learning in Dynamic Environments
Source: Front Neurorobot. 2022 Apr 29;16:846219. doi: 10.3389/fnbot.2022.846219 (PMC9100780; doi:10.3389/fnbot.2022.846219)
Supplement: Supplementary file 1 [file Data_Sheet_1.pdf]

Avoiding Catastrophe: Active Dendrites Enable Multi-Task Learning in Dynamic Environments. Supplementary Materials

1 MULTITASK REINFORCEMENT LEARNING

1.1 Additional Experiments

We ran a number of control experiments that test variations of our Active Dendrites Network in order to better understand the impact of architectural decisions (Table S1). For these experiments we use the hyperparameters as presented in Table 2. The average success rate for each architecture is averaged over 5 independent trials across the last 500,000 environment steps of training. For comparison, the Active Dendrites Network we present in Table 2 achieved an average success rate of **87.5%**.

	1-Layer	3-Layer	1-Seg.	20-Seg.	2-Modulated
Network Details					
Hidden Sizes	1×[4,000]	3×[2,000]	2×[2,800]	2×[2,800]	2×[2,800]
Num. Dendritic Segments	10	10	1	20	10
Hidden Layers Modulated	[✓]	[×, ×, ✓]	[×, ✓]	[×, ✓]	[✓, ✓]
Total Non-zero Parameters	558,804	7,483,404	7,197,964	7,729,964	7,729,964
Average Success Rate	**58.9%**	**87.7%**	**85.6%**	**89%**	**82.5%**

Table S1: The architecture details for each additional multi-task RL experiment. The ***n*-Layer** experiments correspond to networks with n hidden layers and modulated by 10 dendrites in the final hidden layer. The ***n*-Seg.** experiments correspond to networks with 2 hidden layers and modulated by n dendrites in the final hidden layer. The **2-Modulated** experiment uses a 2 hidden layer network, where both hidden layers are modulated by 10 dendrites each.

Our Active Dendrites Network experiments suggest that using more than a single hidden layer and modulating only the final hidden layer produces the best results. In addition, modulation with more dendritic segments than the number of tasks results in marginal increases in success, while less dendritic segments than the number of tasks produces a marginal decrease in success. These findings are consistent with those observed in our continual learning experiments.

2 CONTINUAL LEARNING

2.1 Understanding Parameters in the Model

One interesting issue relates to the size of the Active Dendrites Network and the total number of non-zero parameters. In addition to feedforward weights, our neurons have weights associated with each dendritic

segment. In most of our experiments the number of dendritic segments is set to $\mathcal{T}$, the number of tasks (see Section 2.3 for results with a fixed number of dendritic segments). We can calculate the number of weights in each hidden layer l of the network as follows. Let p be the size of the prototype vector, n_l be the number of units in layer l, s^F be the weight sparsity for the feedforward weights and s^D the weight sparsity for dendritic weights. The total number of weights in layer l is then:

$$W_l = \underbrace{\left(\left(1 - s^F\right) n_l + 1\right) n_{l-1}}_{\text{Feedforward weights \& biases}} + \underbrace{\left(1 - s^D\right) p\mathcal{T} n_l}_{\text{Dendritic segments}}$$

The first term represents the total number of weights in the feedforward portion (including a bias). The second term represents the number of weights in the dendritic segments. In our implementation, $s^F = 0.5$, and $s^D = 0$ (i.e., dendritic weights are fully dense).

In addition to these weights we also store $\mathcal{T}$ prototypes, each of which has the same size as the input vector. Although these are not learned through backpropagation, they are determined from the training data and should be included in the parameter count. In permutedMNIST the input vector size is $n_0 = 784$, leading to a total of $\mathcal{T} \times 784$ additional values for the prototypes.

The number of dendritic weights quickly dominates all other parameters as the number of tasks increases (Table S2, middle column). At first glance, the implication is that the number of parameters in our 100-task network is far greater than the number of parameters in the comparison networks. However notice that the dendritic segments do not receive the input. The dendritic segments determine a context-dependent scale factor per neuron, based only on one of $\mathcal{T}$ possible context vectors. This scale factor is learned during training but then is static during testing.

Since there is a small fixed pool of $\mathcal{T}$ prototype vectors, a simple post-processing step can replace the weights with a smaller identical system. The output of an Active Dendrites Neuron is:

$$\hat{y} = \left(\boldsymbol{w}^\top \boldsymbol{x} + b\right) \times \sigma\left(\max_j \boldsymbol{u}_j^\top \boldsymbol{c}\right)$$

During testing $\sigma\left(\max_j \boldsymbol{u}_j^\top \boldsymbol{c}\right)$ is constant for each vector $\boldsymbol{c}_i$. The equation can be re-written as:

$$\hat{y} = \left(\boldsymbol{w}^\top \boldsymbol{x} + b\right) d_i$$

where $d_i = \sigma\left(\max_j \boldsymbol{u}_j^\top \boldsymbol{c}_i\right)$, $0 < i \leq \mathcal{T}$. For any given test input, we can select the nearest prototype vector i and use the appropriate scale factor d_i. The total number of *effective parameters* in layer l is thus reduced to:

$$W_l' = \left(\left(1 - s^F\right) n_l + 1\right) n_{l-1} + \mathcal{T} n_l$$

Note that in the experiments reported here, with a small fixed number of context vectors, it is actually possible to learn d_i directly via backpropagation. In this case the number of non-zero parameters would be identical to the number of effective parameters, even during training. We did not implement this as it would also limit the flexibility of the overall architecture and disallow future scenarios where the context vector

changes dynamically per input. In Table S2 we list the total and effective number of parameters for the Active Dendrites Network in comparison to some of the other networks. Note that the Active Dendrites Network has substantially fewer effective parameters than any of the other networks.

Network	Tasks	Non-Zero Parameters	Effective Parameters
Active Dendrites Network	10	35,034,794	2,963,114
Active Dendrites Network	100	324,119,114	3,402,314
3-layer MLP	10 or 100	5,824,522	5,824,522
10-layer MLP	10 or 100	35,198,986	35,198,986
XdG	10 or 100	5,592,010	5,592,010

Table S2: The total and effective number of parameters for Active Dendrites Networks as compared to some of the other networks. Note: that XdG requires a mapping from task ID to sub-networks that is not incorporated in the table.

In our previous work (Hawkins and Ahmad, 2016) we used extremely sparse dendritic weights ($> 99\%$ sparsity). These weights were dynamically determined during the learning process by sampling from components of the context vector. Consistent with the biology of active dendrites, the number of weights per segment was limited to a small constant (such as 30). Implementing sparse dendritic weights in the context of deep learning systems is an important future research area for Active Dendrites Networks.

2.2 Number of Clusters Formed when Inferring Prototypes

In our continual learning experiments, when inferring the context vector while training via clustering, we used a significance threshold of $p = 0.9$. We chose this value arbitrarily, and can further improve our results by incorporating p as a model hyperparameter. Assuming that the prototype vector for each permutedMNIST task is sufficiently different, we found that our method arrives at a "sensible" number of prototypes (i.e., not too few nor too many clusters/prototypes as compared with the number of tasks). Figure S1 shows the average number of clusters formed as a function of the number of permutedMNIST tasks we trained an Active Dendrites Network on.

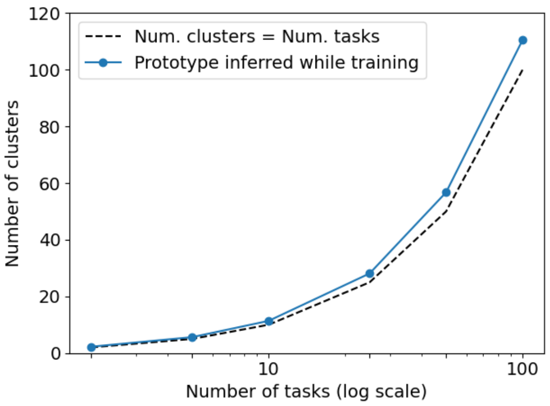

Figure S1: The average number of clusters found by the clustering procedure (described by Algorithms 1 and 2 as a function of the number of permutedMNIST tasks. All results are averaged over 8 independent trials.

2.3 Active Dendrites Network with a Fixed Number of Parameters

In our continual learning experiments we mentioned that for any given number of permutedMNIST tasks, a single Active Dendrites Neuron has the same number of dendritic segments as tasks. The total number of learnable, non-zero parameters in that scenario grows linearly with the number of tasks. (Table S2 lists each model's parameter count.) Although the number of effective parameters is far smaller than the actual parameter count (see Section 2.1), we also tested learning 100 tasks in sequence with a fixed 10 dendritic segments per neuron. This network maintains a constant 35 million non-zero parameters (same as a 10-layer MLP) independent of the number of tasks. As Figure S2 shows, our modified network achieves 78.5% accuracy on 100 tasks, close to the network with 100 dendritic segments.

Why does an Active Dendrites Network not suffer from a severe drop in accuracy with significantly fewer dendritic segments for a large number of tasks? We hypothesize that since the dendritic segments are dense and prototype context vectors are sparse (as most pixels in an MNIST image are black), a single segment can learn to identify multiple context vectors, and thus there can be far fewer dendritic segments than unique context vectors.

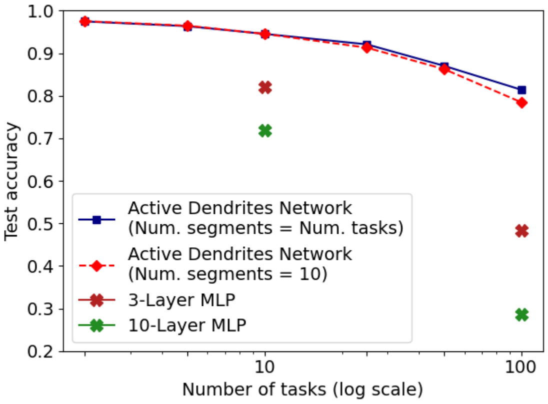

Figure S2: Continual learning accuracy on permutedMNIST: an Active Dendrites Network with the same number of dendritic segments per neuron as the number of tasks (blue), and one with exactly 10 dendritic segments per neuron (red). We also included 3- and 10-layer MLPs on 10 and 100 tasks. All results are averaged over 8 independent trials.

REFERENCES

Hawkins, J. and Ahmad, S. (2016). Why neurons have thousands of synapses, a theory of sequence memory in neocortex. *Frontiers in Neural Circuits* 10, 1–13. doi:10.3389/fncir.2016.00023
